# Metagenomic Analysis Reveals Microbial Community Structure and Metabolic Potential for Nitrogen Acquisition in the Oligotrophic Surface Water of the Indian Ocean

**DOI:** 10.3389/fmicb.2021.518865

**Published:** 2021-02-18

**Authors:** Yayu Wang, Shuilin Liao, Yingbao Gai, Guilin Liu, Tao Jin, Huan Liu, Lone Gram, Mikael Lenz Strube, Guangyi Fan, Sunil Kumar Sahu, Shanshan Liu, Shuheng Gan, Zhangxian Xie, Lingfen Kong, Pengfan Zhang, Xin Liu, Da-Zhi Wang

**Affiliations:** ^1^BGI-Shenzhen, Shenzhen, China; ^2^Department of Biotechnology and Biomedicine, Technical University of Denmark, Kongens Lyngby, Denmark; ^3^BGI Education Center, University of Chinese Academy of Sciences, Beijing, China; ^4^State Key Laboratory of Marine Environmental Science, College of the Environment and Ecology, Xiamen University, Xiamen, China; ^5^Third Institute of Oceanography, Ministry of Natural Resources, Xiamen, China; ^6^BGI-Qingdao, BGI-Shenzhen, Qingdao, China; ^7^State Key Laboratory of Agricultural Genomics, BGI-Shenzhen, Shenzhen, China

**Keywords:** microbe, *Prochlorococcus*, metagenome, nitrogen metabolism, Indian Ocean

## Abstract

Despite being the world’s third largest ocean, the Indian Ocean is one of the least studied and understood with respect to microbial diversity as well as biogeochemical and ecological functions. In this study, we investigated the microbial community and its metabolic potential for nitrogen (N) acquisition in the oligotrophic surface waters of the Indian Ocean using a metagenomic approach. Proteobacteria and Cyanobacteria dominated the microbial community with an average 37.85 and 23.56% of relative abundance, respectively, followed by Bacteroidetes (3.73%), Actinobacteria (1.69%), Firmicutes (0.76%), Verrucomicrobia (0.36%), and Planctomycetes (0.31%). Overall, only 24.3% of functional genes were common among all sampling stations indicating a high level of gene diversity. However, the presence of 82.6% common KEGG Orthology (KOs) in all samples showed high functional redundancy across the Indian Ocean. Temperature, phosphate, silicate and pH were important environmental factors regulating the microbial distribution in the Indian Ocean. The cyanobacterial genus *Prochlorococcus* was abundant with an average 17.4% of relative abundance in the surface waters, and while 54 *Prochlorococcus* genomes were detected, 53 were grouped mainly within HLII clade. In total, 179 of 234 *Prochlorococcus* sequences extracted from the global ocean dataset were clustered into HL clades and exhibited less divergence, but 55 sequences of LL clades presented more divergence exhibiting different branch length. The genes encoding enzymes related to ammonia metabolism, such as urease, glutamate dehydrogenase, ammonia transporter, and nitrilase presented higher abundances than the genes involved in inorganic N assimilation in both microbial community and metagenomic *Prochlorococcus* population. Furthermore, genes associated with dissimilatory nitrate reduction, denitrification, nitrogen fixation, nitrification and anammox were absent in metagenome *Prochlorococcus* population, i.e., nitrogenase and nitrate reductase. Notably, the *de novo* biosynthesis pathways of six different amino acids were incomplete in the metagenomic *Prochlorococcus* population and *Prochlorococcus* genomes, suggesting compensatory uptake of these amino acids from the environment. These results reveal the features of the taxonomic and functional structure of the Indian Ocean microbiome and their adaptive strategies to ambient N deficiency in the oligotrophic ocean.

## Introduction

The Indian Ocean is one of the largest oligotrophic water bodies, which covers approximately one-fifth of global ocean (Eakins and Sharman, 2010; Wei et al., 2019). As the warmest ocean on the earth, the Indian Ocean possesses unique biophysical properties that strongly influence the diversity and performance of its biota (Massana et al., 2011; Williamson et al., 2012; Díez et al., 2016; Thompson et al., 2017). However, the Indian Ocean is one of the least studied oceans regarding microbial community structure, functional capacity and the potential linkage of microbial taxa and environmental conditions, when compared with other oceans (Venter et al., 2004; Joint et al., 2011; Sjöstedt et al., 2014; Mende et al., 2017; Li Y. et al., 2018). A recent study on biodiversity and spatial distribution of bacteria in the water column of the eastern Indian Ocean has shown that Cyanobacteria and Actinobacteria are more predominant in the upper ocean while Alphaproteobacteria occur more frequently in the deeper layer (Wang et al., 2016). However, a study in the South Indian Ocean has indicated that Gammaproteobacteria dominate the microbial community and their potential functionality is shaped by the depth-related environmental parameters of the Agulhas Current (Phoma et al., 2018). Metagenomic analysis of the picocyanobacterial community in the equatorial waters of the Indian Ocean reveals that the genera *Prochlorococcus* and *Synechococcus* comprise 90% of the cyanobacterial reads (Díez et al., 2016). *Prochlorococcus* populations in the ocean have been defined with high light-adapted (hereafter HL) populations and low light-adapted (hereafter LL) populations, where *Prochlorococcus* HLIIA ecotype is abundant in the *Prochlorococcus* community in the Indian Ocean (Farrant et al., 2016). All together, these studies have provided a snapshot of the microbial diversity and geographical distribution in some specific areas of the Indian Ocean, but knowledge on the overall microbial taxonomic structure and functional capacity, and their influencing factors are still very limited.

Nitrogen (N) is an essential macronutrient limiting microbial growth and proliferation in the ocean, especially in the oligotrophic oceanic regimes (Zehr and Wad, 2002; Moore et al., 2013). Ambient N deficiency affects N assimilation, carbon fixation, photosynthesis, pigment and lipid accumulation of microbes (Schwarz and Forchhammer, 2005). Typically, a broad diversity of prokaryotes from Proteobacteria, Firmicutes, Verrucomicrobia, Planctomycetes, Acidobacteria, Chloroflexi, and Chlorobia can use dissolved inorganic N (DIN), such as nitrate, nitrite and ammonia, and these nutrients are sufficient to support microbial growth in coastal and upwelling areas (Li Y.-Y. et al., 2018; Pajares and Ramos, 2019). However, these N nutrients are extremely low in quantity throughout much of the surface oligotrophic ocean (Moore et al., 2013) and cannot support microbial growth. Instead, microbes have evolved diverse adaptive strategies to ambient N deficiency, for example, utilizing small but rapidly cycling dissolved organic nitrogen (DON), such as free amino acids, amines and urea as N source for cell growth and proliferation, indicating the essential roles of DON in maintaining microbial communities in the oligotrophic ocean (Wheeler and Kirchman, 1986; Zubkov et al., 2003; García-Fernández et al., 2004). However, little is known concerning nitrogen acquisition by microbes in the oligotrophic Indian Ocean (Kumar et al., 2009; Díez et al., 2016; Qian et al., 2018; Baer et al., 2019), which impedes our understanding of the mechanisms underlying the adaptive strategies of microbes to ambient N deficiency.

**FIGURE 1:**
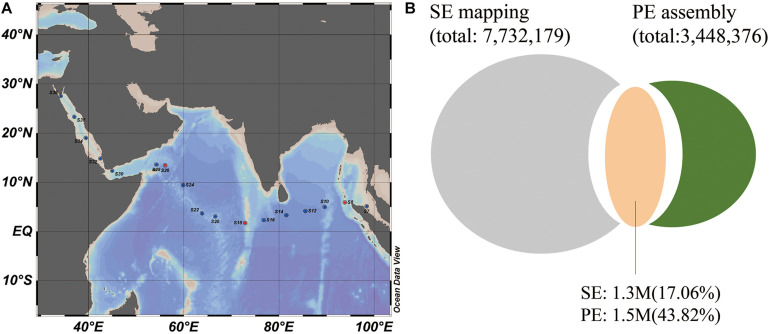
Sampling locations and the microbial genes found in the Indian Ocean. **(A)** Sampling stations across the Indian Ocean and Red Sea. **(B)** The number of shared genes (orange) between the 17 SE sample gene set (gray) and three PE sample assembled gene set (green). 1.3 M and 1.7 M genes from two gene sets were shared, which occupied 17.06 and 48.3% of the gene numbers in two gene sets, respectively.

Metagenomic studies of the global ocean advance our understanding of diversity, evolution and functional potential of natural microbial communities (Sunagawa et al., 2015; Li Y. et al., 2018). Distribution of microbial diversity and biogeochemistry are structured largely by environmental gradients such as light, temperature, oxygen, salinity, and nutrients (Thompson et al., 2017). In this study, we applied a metagenomic approach to investigate microbial communities in the oligotrophic surface waters of the Indian Ocean, from the Andaman Sea in the east to the Red Sea in the west, and characterized their nitrogen acquisition strategies based on a 9.5 million (M) microbial gene set (Figure 1 and Supplementary Figure S1). We paid particular attention to the predominant cyanobacterial genus *Prochlorococcus* and their nitrogen assimilation strategies due to its significant contribution to the stability, resilience and function of the marine ecosystem. This study expands our understanding of the microbial community and their metabolic potentials in the Indian Ocean, and provides fundamental metagenomic data for further microbial studies in the Indian Ocean, which is a significantly understudied realm.

## Materials and Methods

### Sampling and DNA Extraction

A total of 17 surface seawater samples (S6–S38, 5 m depth) were collected from the outlet of profiling CTD (Seabird SBE21) during the 26th cruise of China Ocean Mineral Resources R & D Association (COMRA^1^) on the R/V DayangYihao in May 2012. The sampling stations ranged across the Indian Ocean, from the Andaman Sea in the east to the Red Sea in the west (Figure 1A). Samples from the Red Sea were specifically referred to as S32, S34, S36, and S38. For each sample, more than 200 l of seawater was prefiltered using a GF/A glass fiber membrane (1.6 μm, 142 mm filter diameter, Whatman) with a Flojet Pump (04300242A) and then collected on a Supor polyethersulfone membrane (0.2 μm, 142 mm filter diameter, Pall). The membranes were placed in 50 ml tubes and immediately frozen in liquid nitrogen, and then stored at −80°C until further analysis. DNA extraction was performed using the PowerSoil DNA isolation kit (QIAGEN, United States) according to the instructions. Environmental parameters were also monitored, including temperature, salinity, pH and inorganic nutrients listed in Supplementary Table S1. Concentrations of phosphate, silicate, ammonium, nitrite and nitrate as well as chlorophyll-*a* concentration were measured in the laboratory according to the national standard protocol for investigation of chemical elements of seawater titled “Specifications for oceanographic survey-Part 4: Survey of chemical parameters in seawater” (GB/T 12763.4-2007, 2007).

### DNA Sequencing on BGISEQ-500 and HiSeq4000

All 17 DNA samples were sequenced using a 50 bp single-ended (SE) sequencing strategy on a BGISEQ500 platform (Beijing Genomics Institute, Shenzhen). Firstly, the DNA sample was fragmented by ultrasound on Covaris E220 (Covaris, Brighton, United Kingdom). Then 400–700 bp DNA fragments were recovered to construct a library for each sample according to standard instructions (Fang et al., 2017). Three barcoded libraries were pooled together with equal amounts to make DNA nanoballs (DNB). Each DNB was loaded into one lane for sequencing on the BGISEQ-500 sequencer with the SE50 mode. The sequence images were base-called by the software Zebra call (base calling software developed for BGISEQ-500). A total of 12.81 billion raw reads were produced by the BGISEQ-500 platform, with a read length of 50 bp.

Since long and pair-end (PE) reads are more useful for metagenome assembly, three representative local samples were collected from the stations of S8 (E 93°49.228′, N 5°54.323′), S18 (E 72°52.169′, N 1°42.848′) and S26 (E 56°5.870′, N 13°26.297′) located at the east, central and west regions of the Indian Ocean, and were selected for in-depth sequencing using a 150 bp PE strategy on Hiseq4000 platform (Illumina). A total of 749.03 M reads were generated with an average 249.6 M reads for each sample.

### Construction of the Gene Catalog

After a quality check and pre-processing by SOAP nuke (v1.5.3) (Chen et al., 2017), all clean reads of BGISEQ were aligned against the Ocean Microbiome Reference Gene Catalog (OM-RGC) (Sunagawa et al., 2013) using bowtie2.2.6 (Langmead and Salzberg, 2012) with parameters: bowtie2 –q –phred33 –sensitive –mixed –no-discordant -p 20 -k 200 -x. Then microbial genes mapped by unique reads were considered to be present in the samples. On average, 72.68% of BGISEQ SE reads were mapped to the OM-RGC, resulting in an average of 2.92 M genes in each sample (Supplementary Table S2). All the genes identified from 17 samples were merged together and then duplicates were removed, generating a 7.7 M unique gene set. We named this set the 17 SE sample gene set (Supplementary Table S2).

After quality control, the Illumina PE reads of three representative local samples (S8, S18, and S26) were *de novo* assembled into contigs using MegaHit (v1.0) with parameters: -t 20 -min-contig-len 500 –presets meta-large –min-count 5 (Li et al., 2016). A total of 23.04% of the Illumina PE reads were assembled into 3.26 M contigs (= 500bp). GeneMarkS (version 2.7) (Besemer et al., 2001) was used to predict the genes using the parameter: gmhmmp -m MetaGeneMark_v1.mod in contigs obtained from each sample. The length of the predicted genes was no less than 150 bp. A non-redundant gene catalog (3.4M) was built by clustering all predicted genes using CDHIT (version 4.6.5) (Li and Godzik, 2006) with the following parameters: 95% identity and 90% coverage. The 3.4 M assembled gene set (called three PE sample gene set) was then compared with the 7.7 M gene set (17 SE sample gene set) to identify the overlapping genes using CDHIT with the same cutoff as above. As the shared genes accounted for a low proportion (Figure 1B) of the two gene sets, we merged these two gene sets together. We then removed the redundancy to generate a more complete gene set for the Indian ocean samples.

### Taxonomic and Functional Annotation

Taxonomic and functional assignment of genes of the complete gene set was performed. Taxonomic assignments were performed by aligning protein sequence to the NCBI-NR database using DIAMOND (version 0.8.22) (Buchfink et al., 2015). The top alignment hits were retained according to the criteria of coverage ≥ 80%, identity ≥ 65%, and *e*-value ≤ 1e-5 as previous studies described (Qin et al., 2010; Li et al., 2014). Then, the taxonomic annotation of each gene was determined by the lowest common ancestor (LCA)-based algorithm implemented in MEGAN (Huson et al., 2007). Functional annotations were made by aligning the protein sequence against eggNOG (version 4.5) (Huerta-Cepas et al., 2016b) and KEGG (version.81) database by the highest scoring annotated hit(s) containing at least one high-scoring pair (HSP) scoring over 60 bits by DIAMOND.

### Statistical Analyses

The relative abundances of genes were calculated based on the unique mapping reads as previously described (Qin et al., 2012). Firstly, the read counts from each gene were normalized by dividing with the gene length to calculate gene abundance. Then the relative abundance of each gene in samples were generated by dividing with the sum of abundance of all genes. Each KO abundance was generated by summing up the relative abundance of genes annotated to the same KO. The taxonomic profile was constructed using the same method. The meta-genes (unigenes) in the metagenomic gene set assigned to the genus of *Prochlorococcus* were extracted and the sum of their abundance in each sample was determined as the abundance of *Prochlorococcus*. The difference in abundance of *Prochlorococcus* between the central ocean and the coast samples, and the differences in dissolved inorganic nitrogen (DIN) concentration between the central ocean samples and coast samples were checked using *t*-test (basic function in R version 3.5.3). The clustering analysis based on physicochemical properties of all samples was performed using function pheatmap () in R package (version 3.5.3). To determine the shaping factors causing the variation in microbial communities among samples, the environmental variables were included in a redundancy analysis (RDA) model using the function rda () in “vegan” R-package, which was then reduced by the step-function in R according to the Akaike information criterion (AIC). The correlations of the relative abundance of *Prochlorococcus* with nitrate, nitrite and ammonium concentrations were separately calculated using Spearman’s rank correlation test using the function cor. test () in R package (version 3.5.3).

### Analysis of N Metabolism and Amino Acids Biosynthesis

The KOs involved in N metabolism were extracted from the merged gene set to analyze functional capacity of the Indian Ocean microbiome. The KOs involved in N metabolism and the annotated *Prochlorococcus* data were extracted to explore the N assimilatory pathways of the *Prochlorococcus* population in the Indian Ocean. The key enzymes were also manually annotated using the Pfam database to validate the existence of functional domain. All KOs in map01230-Biosynthesis of amino acids were extracted to explore biosynthesis reactions of amino acids. The KOs functionally annotated with “transporter” were extracted from the metagenome of *Prochlorococcus* population and the identified single *Prochlorococcus* genome respectively to predict the uptake model of amino acids from the environment. The single *Prochlorococcus* genome was identified based on 201 published *Prochlorococcus* genomes and the Indian Ocean metagenome sequencing data, and the detailed process was described below.

### Phylogenetic Tree of *Prochlorococcus* Genomes

A total of 201 *Prochlorococcus* genomes including 27 known ecotype genomes (Biller et al., 2014) were downloaded from NCBI (Supplementary Table S3). The gene prediction of each genome was performed using GeneMarkS (version 2.7) (Besemer et al., 2001) with the parameter: gmhmmp -m Prochlorococcus_prefix.mod. All genes were merged together to construct a pangenome. Then the SE reads from each sample were mapped against the pangenome using bowtie2.2.6 to identify *Prochlorococcus* genes. If the portion of gene numbers mapped by reads was more than 50%, the Prochlorococcus strain was considered to be present in the sample. The abundance of each identified gene was calculated by dividing the mapping reads with the gene length. The abundance of the identified *Prochlorococcus* was represented by the average abundance of their identified genes. The same 40 single copy genes (SCGs) (Mende et al., 2013) from the 55 identified strains and 25 known ecotype strains were predicted by fetchMG (Kultima et al., 2012; Sunagawa et al., 2013). Then multiple alignments of 40 SCGs were performed using Mafft (Kazutaka and Daron, 2013). Here we selected 25 of 27 known ecotype strains as reference because we could not extract a full number of SCGs genes from the remaining two strains genomes. A phylogenic tree was constructed using ETE3 and the standard FastTree workflow (Price et al., 2010; Huerta-Cepas et al., 2016a).

To evaluate the diversity of *Prochlorococcus* population in the global ocean, 40 SCGs were extracted from 201 reference genomes, OM-RGC and the Indian Ocean gene set by fetchMG. Among all the screened genes, the gene family COG0172 (*Seryl-tRNA synthetase*) showed the highest number, therefore, it was selected to construct the phylogenetic tree. Only the genes having a sequence length higher than 800 bp were retained in the tree.

## Results

### Microbial Gene Capacity in the Indian Ocean

The locations of the 17 sampling stations along an east-west transect in the Indian Ocean (12°S, 96°E to 4°S, 39°E) are shown in Figure 1A. In total, 12.68 billion high quality SE reads were generated, resulting in an average of 37.16 Gb data per sample. Here, the microbial functional genes were identified by mapping these SE reads to the *Tara* Oceans gene set (OM-RGC, 40M genes). An average of 3 M genes per sample were identified with a 72.68% mapping rate. The reads of three PE sequenced samples were also mapped to OM-RGC and 94.90% of identified genes by PE reads were covered by SE reads (Supplementary Figure S2), indicating that the SE data was sufficient to map reference genes in this study. By merging all identified microbial genes of the 17 SE sequenced samples together, a 17 SE sample gene set was generated, comprising 7.7 M non-redundant genes (Supplementary Table S2).

To mine more specific microbial genes in the Indian Ocean surface water, a 3.4 M gene set based on *de novo* assembly was generated using three PE sequenced samples, which obtained 51.95–52.16% mapping rate in individual samples. The shared genes between the 17 SE sample gene set and three PE sample assembled gene set accounted for an average of 17.06 and 43.82% of gene abundance in the SE mapping gene set and PE assembly gene set (Figure 1B), suggesting that many novel genes could be explored by *de novo* assembly from local samples. So, a new complete microbial gene set (9.5M) from the Indian Ocean surface waters was generated by the combination of OM-RGC mapping and *de novo* assembly methods. Based on the new gene set, many more genes could be identified with higher reads mapping rate in each sample.

### Taxonomic and Functional Variations in the Indian Ocean

**FIGURE 2:**
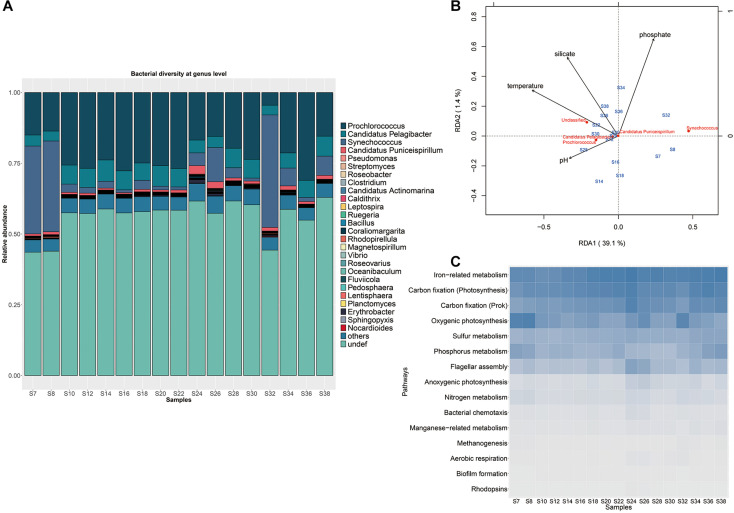
Microbial communities in the surface waters of the Indian Ocean. **(A)** Microbial community structure with abundant *Prochlorococcus* and *Synechococcus*. The samples from stations S7, S8, and S32 near the coast with distinct taxonomic compositions distinct from the other samples. **(B)** Redundancy analysis of bacterial community structure and environmental variables. **(C)** Distributions of typical metabolism pathways among different sampling stations.

A large proportion of genes (51.91%) in the complete gene set belong to bacteria, while 7.96%, 2.00% and 1.10% were annotated to virus, eukaryote and archaea, respectively (Supplementary Figure S3A). More than 69.51% of bacterial sequences could be annotated at the phylum level. Proteobacteria and Cyanobacteria dominated the microbial community by occupying an average 37.85 and 23.56% of total bacterial sequences of the Indian Ocean, respectively, followed by Bacteroidetes (3.73%), Actinobacteria (1.69%), Firmicutes (0.76%), Verrucomicrobia (0.36%), and Planctomycetes (0.31%) (Supplementary Figure S3B). Totally, 684 bacterial genera were found in the Proteobacteria phylum, of which the genus *Candidatus pelagibacter* was dominant with an average of 12.03% of relative abundance (Supplementary Table S4). The genera in the Bacteroidetes phylum presented similar relative abundances, such as the dominant genera *Fluviicola (1.53%* of relative abundance) and *Flavobacterium* (0.97% of relative abundance). The Actinobacteria were represented mainly by *Streptomyces* (5.41% of relative abundance) and *Candidatus actinomarina* (4.81% of relative abundance). The Firmicutes were mainly composed of *Clostridium* (8.80% of relative abundance) and *Bacillus* (8.83% of relative abundance). The genera *Coraliomargarita* (25.89% of relative abundance), *Pedosphaera* (11.05% of relative abundance) and *Verrucomicrobium* (5.24% of relative abundance) dominated the phylum of Verrucomicrobia. While *Rhodopirellula* (25.94% of relative abundance) and *Planctomyces* (12.13% of relative abundance) were the abundant groups of Planctomycetes. Among them, the photosynthetic cyanobacterial taxa such as *Prochlorococcus* and *Synechococcus* were the most abundant in each sample, comprising 86.11% of total cyanobacterial sequences (Figure 2A and Supplementary Table S4). Particularly, *Synechococcus* with higher relative abundance (average 25.9% of total bacterial sequence) predominated in the samples collected from S7, S8, and S32 stations close to the coastal areas with relatively high DIN concentration while *Prochlorococcus* dominated with an average 17.4% relative abundance in the middle area with low DIN concentration (Supplementary Table S1, *t*-test, *P*-value < 0.05) (Figure 2A). Besides, no obvious difference in microbial composition between the Red Sed and other locations of the Indian Ocean. Notably, the abundance of *Prochlorococcus* was negatively correlated with the abundance of *Synechococcus* across the transect (*P*-value = 2.2e^–16^, Spearman’s rank correlation test with the correlation coefficient = −0.88). Although the DIN concentrations of the Indian Ocean surface waters varied among stations, it was much lower than the minimum detection limit and the N/P ratio was less than 8 (Supplementary Table S1), indicating that the Indian Ocean surface water belonged to an oligotrophic water body.

The clustering analysis based on physicochemical properties of all samples showed that the 17 samples were divided into three groups, the three Red Sea samples (S34, S36, and S38) were grouped together with the sample S30 from the west of the Indian Ocean, the Red Sea sample S32 was clustered with the samples S18, S22, S24, S26, which were located in the middle of the Indian Ocean, while the other remaining samples were grouped together (Supplementary Figure S4). These results demonstrated that the physicochemical properties of the west region of the Red sea was distinguishable from the Indian Ocean. RDA was performed to discern the relationship between microbial community structure and environmental parameters such as concentrations of phosphate, silicate, ammonium, nitrite, nitrate, as well as temperature, salinity and pH (Supplementary Table S1). A model describing the environmental parameters that were significantly correlated with microbial composition was selected based on Akaike information criterion, indicating that temperature, phosphate, silicate and pH rather than ammonium, nitrite, nitrate and salinity had significant effect on the microbial composition. The two axes could explain 40.5% of the overall variance in community structure with an adjusted *R*^2^ (0.56), showing a remarkable correlation. The parameters of temperature, phosphate, silicate and pH drove the composition variance and separated samples into different groups, of which temperature was the most important and could explain the most variation in response variables (Figure 2B). It was found that the three western Red sea samples were grouped in the upper right of the RDA plot, and were mainly associated with concentrations of phosphate and silicate. But most of the Indian Ocean samples (except samples S7 and S8 near the coast) were in the lower left and were associated with temperature and pH. These results indicated that the variation among microbial communities in the Red Sea and in the Indian Ocean were driven by different environmental factors. In addition, the dominant genus *Prochlorococcus* was positively associated with temperature and pH in the multivariate analysis, while the genus *Synechococcus* was negatively associated with pH. Interestingly, the concentrations of ammonium, nitrate and nitrite were not associated with the variation of the whole microbial community. However, the abundance of *Prochlorococcus* presented a remarkably negative correlation with nitrite concentration (*P*-value < 0.01, Spearman’s rank correlation test), but not with the concentrations of ammonium, nitrate, phosphate and other factors, indicating that nitrite impacted *Prochlorococcus* although it had no effect on the whole microbial composition of the Indian Ocean.

Variations of microbial functional composition were examined among stations. Overall, only 24.3% of genes but 82.6% of KOs were co-existed in all samples, indicating a high level of gene diversity and functional redundancy across the Indian Ocean. These shared KOs mainly contributed to metabolic function (carbohydrates, amino acids, nucleotide, cofactors and vitamins, lipid, energy metabolism), genetic information processing (replication and repair), secondary metabolite biosynthesis (glycan, terpenoids, and polyketides), and signal transduction, and the majority of them corresponded to housekeeping functions. The abundances of 15 typical pathways were calculated using a total abundance of marker KOs divided by the number of KOs as described in a previous study (Sunagawa et al., 2015). All samples showed a similar variation pattern in functional composition (Figure 2C). Function modules associated with iron-related metabolism, carbon fixation (photosynthesis), carbon fixation (Prok), oxygenic photosynthesis, sulfur and phosphorus metabolism with high abundances co-existed in all samples. In addition, other processes such as nitrogen metabolism, methanogenesis, manganese–related metabolism, flagellar assembly, bacterial chemotaxis, biofilm formation, aerobic respiration and anoxygenic photosynthesis with low abundances were prevalent in each sample.

### *Prochlorococcus* Genomes in the Indian Ocean and the Global Ocean

**FIGURE 3:**
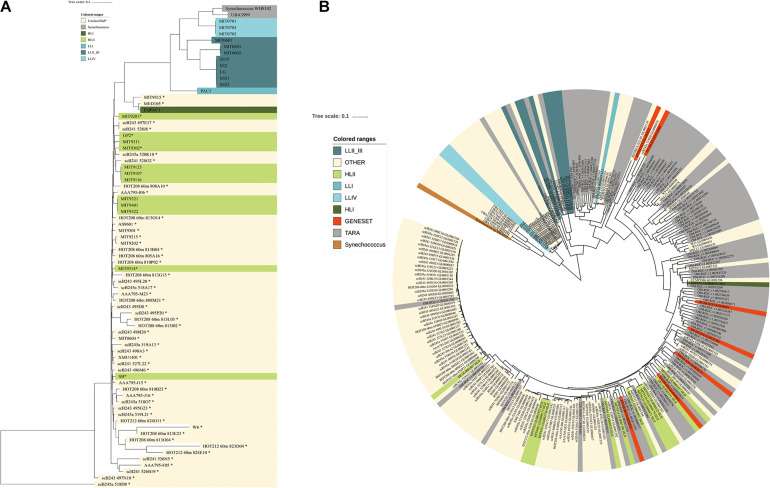
Phylogenetic tree based on single copy genes from the 54 detected strains in the Indian Ocean and *Prochlorococcus* sequences from the global ocean, respectively. **(A)** Phylogenetic tree from the detected strains in our data set (names in bold with^∗^) and representative *Prochlorococcus* genomes was constructed based on 40 SCGs. Phylogenetic clade affiliations are represented by different colors; HLII, light green; HLI, dark green; LLI, median blue; LLII_III, dark blue; LLIV, light blue; *Synechococcus*, gray; unclassified strains, yellow. **(B)** The Phylogenetic tree of *Prochlorococcus* populations in the global ocean based on gene sequences from COG0172. red, sequences identified in assembled gene set; gray, sequences from Tara Ocean gene set; yellow, sequences from *Prochlorococcus* genomes; reference sequences are colored according to their ecotypes.

*Prochlorococcus* an extensively studied photosynthetic bacterial taxon, dominated the microbial community of the Indian Ocean surface water with an average abundance of 14.5% among metagenome sequences (Figure 2A). Using the pangenome of the 201 published *Prochlorococcus* genomes as a reference (Supplementary Table S3), about 14.08% of reads could be mapped to the *Prochlorococcus* gene set, which was very close to the abundance identified in the metagenome data, implying the integrity of the reference data set. Totally, 55 *Prochlorococcus* strains were detected, including 12 cultured strains and 43 uncultured strains. The detections of 55 strains implied the species diversity of *Prochlorococcus* in the surface water of the Indian Ocean. Of these, scB245a_518D8, scB241_528J8, MIT9301, MIT9302, GP2 and MIT9201 were most abundant *Prochlorococcus* strains among the samples (Supplementary Figure S5). Only 5 out of 12 cultured strains are within known HLII ecotype. All of 43 uncultured single-cell amplified genome and metagenome-assembled genomes have no ecotype information. To explore the evolutional relationship of these *Prochlorococcus* strains, phylogenetic clades of all these unknown strains (combined with 25 cultured strains with representative ecotypes) were explored using an alternative method based on 40 SCGs. The phylogenetic tree showed that all the identified genomes were divided into two major known ecotypes: HLI and HLII clades (Figure 3A), in accordance with their niches. Except for the strain UBA3999, which was finally validated to be affiliated to *Synechococcus* as only 70.81% average nucleotide identity (ANI) to *Prochlorococcus* strain MIT0701 and 30 conserved SCGs consistently annotated to *Synechococcus*, 53 of 54 *Prochlorococcus* strains in the Indian Ocean surface waters belonged to the HLII ecotype and only one strain belonged to the HLI ecotype. The ecotype-level community structure was similar across the Indian Ocean, but the relative abundances of *Prochlorococcus* strains varied among different stations. For example, the composition of *Prochlorococcus* strains in the Red Sea samples (S30, S32, S34, and S38) were clustered together and were more similar to the west locations of the Indian Ocean (Supplementary Figure S5).

To further characterize the diversity of *Prochlorococcus* in the global ocean, the 40 SCGs of *Prochlorococcus* genus were fetched from the Tara Ocean gene set, the PE sample assembled gene set and 201 *Prochlorococcus* strains. As the multiple sequence alignment was not suitable for the SCGs from metagenome data, a phylogenetic tree of *Prochlorococcus* was constructed based on one gene, namely COG0172 (*Seryl-tRNA synthetase*) as its highest number in different data sources. This gene is an evolutionarily conserved enzyme which catalyzes the formation of aminoacyl-tRNAs that is used as substrates for ribosomal protein biosynthesis. The *Prochlorococcus* sequences were divided into two main clades, HL and LL (Figure 3B). Of these, 179 of 234 sequences were clustered within the HL clade with similar phylogenic distance and 55 of sequences were clustered within LL clades with different branch distance. It suggested that the *Prochlorococcus* sequences in LL clades evolved more divergently, which might be attributed to the specific features of deep-sea environment.

### Metabolic Potential for Nitrogen Assimilation

**FIGURE 4:**
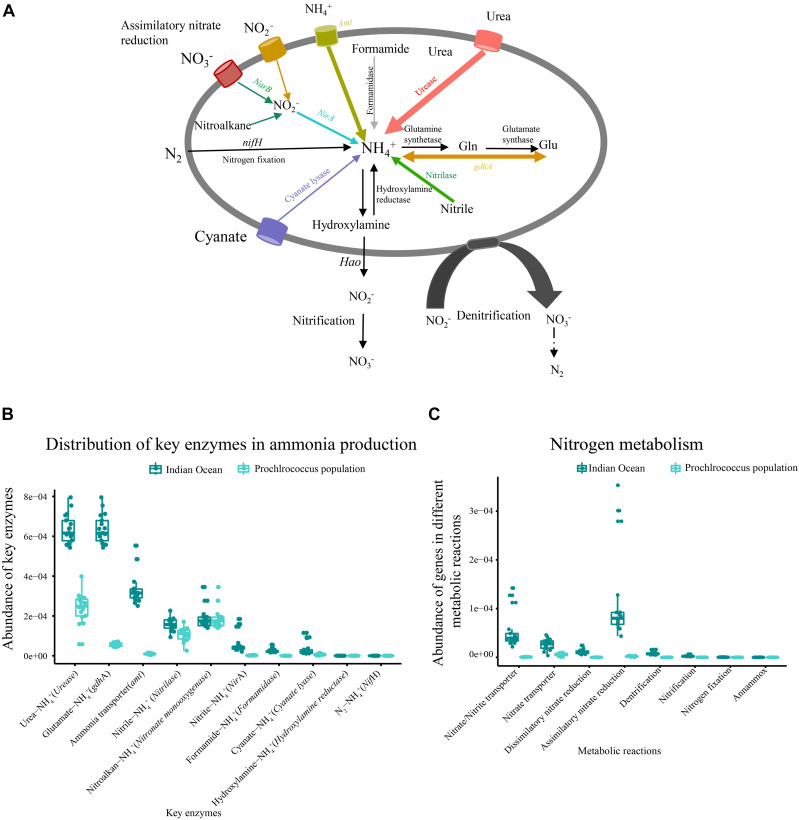
Metabolic potential for N acquisition in the microbial community and metagenomic *Prochlorococcus* population of the Indian Ocean. **(A)** The generic nitrogen assimilation routes (colored line) in the metagenome and *Prochlorococcus* population of the Indian Ocean. **(B)** Abundances of key functional genes involved in ammonia production in the metagenome and *Prochlorococcus* population. **(C)** The functional models of an N metabolism pathway in the metagenome and *Prochlorococcus* population.

The relative abundance of ammonia input-related genes such as urease, glutamate dehydrogenase (*gdhA*) and ammonia transporter (*amt*) were predominant in each sample, followed by nitrilase, ferredoxin-nitrite reductase (*NirA*), cyanate lyase and formamidase (Figures 4A,B). Four metabolic reactions involved in nitrogen metabolism including dissimilatory nitrate reduction, assimilatory nitrate reductase, denitrification and nitrification were detected in our metagenomic dataset (Figure 4C). Although the assimilatory nitrate reduction (*narB*, *nasA*, *narsB, and nirA*) presented high abundance in nitrogen metabolism pathway, it contributed only a small portion to ammonia production. The functional genes related to nitrogen fixation and anammox were not detected in all samples (Figure 4).

To characterize nitrogen acquisition of the *Prochlorococcus* population in the Indian Ocean, all genes involved in nitrogen metabolism from *Prochlorococcus* genus were extracted from the metagenome. *Prochlorococcus* could potentially assimilate nitrogen for growth through six different routes (Figure 4). Three dominant routes were comprised of hydrolyzing urea by ureas, hydrolyzing nitrile to ammonia by nitrilase and converting glutamine into ammonia by *gdhA*. The fourth route was to directly transport extracellular ammonia into intracellular via transporter. The fifth route was to uptake extracellular nitrite and nitrate via transporters, and then catalyze the reduction of nitrite to ammonia by *NirA* and finally be converted into amino acids. The gene encoding nitrate reductase *(NarB*) involved in the reduction of nitrate to nitrite was not identified in the *Prochlorococcus* population. The nitrite could also be converted by oxidation of nitroalkanes using nitronate monooxygenase. The sixth route was to convert cyanate into ammonia by cyanate lyase. Only the metabolic reaction related to assimilatory nitrate reduction was detected in metagenomic *Prochlorococcus* population. The other key enzymes related to dissimilatory nitrate reduction, denitrification, nitrogen fixation, nitrification and anammox were not found (Figure 4C). Collectively, the *Prochlorococcus* population in the Indian Ocean could assimilate urea, ammonia, nitrite, nitroalkane, cyanate, and nitrile as nitrogen sources, but they lost the ability to assimilate nitrate and nitrogen due to the lack of *NarB* and *nifH*.

To validate the results from the metagenome, the abilities of nitrogen metabolism from 54 detected *Prochlorococcus* genomes (including six complete genomes) in the Indian Ocean were evaluated. A similar result was observed in those detected *Prochlorococcus* genomes that the enzyme *nifH* involved in nitrogen fixation was absent but the *NarB* catalyzing nitrate to nitrite was found in 14 strain genomes including two cultured strains (SB, MIT0604) and 12 single-cell amplified genomes (Supplementary Table S5). These results indicated that most of the *Prochlorococcus* strains lost the ability to reduce nitrate, but some of them still maintained the ability to assimilate nitrate and could utilize it as N source. Furthermore, the enzymes involved in assimilation of other nitrogen forms such as cyanate, nitrile, nitrite, and amino acids also differed among strains, suggesting their different survival strategies.

### Amino Acid Biosynthesis of *Prochlorococcus* in the Indian Ocean

The amino acid biosynthesis model was predicted based on the functional enzymes of amino acid biosynthesis and amino acid transporter proteins in the metagenome of *Prochlorococcus* population. Of the 230 functional KOs involved in amino acid biosynthesis, 96 were found in the *Prochlorococcus* population, which contributed to *de novo* biosynthesis of 14 amino acids such as valine, leucine, isoleucine, tryptophan, proline, threonine, cystenine, asparagine, glutamine, aspartic acid, glutamic acid, lysine, arginine, and histidine. However, reaction gaps for the biosynthesis of amino acids existed in six types of amino acids (Table 1). The enzyme *metC* that synthesizes precursor homocysteine of methionine and the *serB* that catalyzes phosphoserine to serine were missing. The enzyme *glyA* that functions bi-directionally transformation of serine and glycine was detected but the bidirection enzyme *glyA* for glycerine and threonine conversion was absent. The enzymes *asdA* and *ALT* for *de novo* biosynthesis of alanine were also absent. Furthermore, biosynthesis reactions of aromatic amino acids tyrosine and phenylalanine were incomplete due to the lack of the enzyme *tyrB* that catalyzes the precursor of tyrosine and converts phenylpyruvate into phenylalanine. Interestingly, the functional genes encoding two types of transporter proteins responsible for importing glycine and alanine into cells were found in the metagenome data of the Prochlorococcus population, suggesting that *Prochlorococcus* absorbed glycine and alanine from the environment. Thus, it could be postulated that serine might be achieved from glycine by *glyA*, and subsequently, converted to cysteine and methionine. The same observation was also found in the genomes of the 54 *Prochlorococcus* strains detected, which further validated the reaction gaps in amino acid biosynthesis of the *Prochlorococcus* population in the Indian Ocean.

**TABLE 1:** Distribution patterns of key enzymes of six amino acids synthesis pathways in *Prochlorococcus* metagenomes from the Indian Ocean.

Amino acids	Key enzymes		Transporters
Met	*metC*	*metH*	–
	–	+	
Ser	*serC*	*serB*	–
	+	–	
Gly	*ltaE* –	*glyA* +	*betT, betS:* choline/glycine/proline betaine transport protein
Ala	*asdA* –	*ALT* –	alanine or glycine cation symporter
Tyr	*tyrB*	*tyrC*	–
	–	+	
Phe	*pheA2*	*tyrB*	–
	+	–	

*metC, cysteine-S-conjugate beta-lyase; metH, 5-methyltetrahydrofolate–homocysteine methyltransferase; serC, phosphoserine aminotransferase; serB, phosphoserine phosphatase; ltaE, threonine aldolase; glyA, glycine hydroxymethyltransferase; asdA, aspartate 4-decarboxylase; ALT, alanine transaminase; tyrB, aromatic-amino-acid transaminase; tyrC, cyclohexadieny/prephenate dehydrogenase; tyrB, aromatic-amino-acid transaminase; pheA2, prephenate dehydratase. +, present; –, absent.*

## Discussion

Metagenomic studies of the global ocean advance our understanding of microbial diversity, community structure, functional potential, the environment influence and biotic interactions (Lima-Mendez et al., 2015; Sunagawa et al., 2015). In this study, we generated a 9.5 M gene set for surface microbes of the Indian Ocean combined with mapped genes from OM-RGC and *de novo* assembled genes from the local samples. Our results showed that most of the microbial genes of surface waters had been embraced by this gene set and could be captured by sample reads. Although the OM-RGC provides an ecosystem-wide data set, which enables ocean microbial genetic diversity accessible for various targeted studies, it does not cover all microbial functional genes owing to the limitation of samples and sampled regions. Therefore, it would be helpful to combine this data with a small set of assembly genes from local samples. Proteobacteria and Cyanobacteria were the abundant phyla in surface waters of the Indian Ocean, and photosynthetic cyanobacterial taxa such as *Prochlorococcus* and *Synechococcus* were dominant in all samples. These results are consistent with the previous studies (Díez et al., 2016; Wang et al., 2016; Phoma et al., 2018). However, variations at the sub-family level classification were observed among different regions of the Indian Ocean, for example, *Synechococcus* was abundant in the stations near the coast but *Prochlorococcus* dominated in the central area of the Indian Ocean, which might be caused by environmental variables. The effects of environmental factors on marine microbial communities have been widely studied (Coutinho et al., 2015). Composition, distribution and metabolic potential of microbes are structured, to a large extent, by environmental gradients in light, temperature, oxygen, salinity and nutrients (Thompson et al., 2017). Globally, temperature is the key environmental factor driving the geographical distribution of microbes in the ocean (Sunagawa et al., 2015). In this study, temperature, phosphate, silicate and pH were important environmental factors regulating microbial distribution in the Indian Ocean. However, the variation of microbial composition in the Red Sea was mainly associated with the concentrations of phosphate and silicate (Figure 2B). It should be noted that the physicochemical properties in the west of the Red Sea differed from the Indian Ocean, resulting in a slight difference in microbial composition. Investigation of microbial adaptation to environmental changes indicated that temperature explains most of the taxonomic and functional variations in the Red Sea, followed by nitrate, chlorophyll, phosphate, and salinity (Thompson et al., 2017). These highly shared KO functions across the samples might confer a buffering capacity for an ecosystem in scenarios of biodiversity loss. The analysis of typical pathways indicated that metabolic processes of microbial community mainly contributed to the production of a diverse array of nutritional substrates such as ferrous/ferric iron, carbohydrate, phosphate, and sulfate. Especially for carbon fixation and oxygenic photosynthesis, they enabled microbes to use organic compounds and molecular oxygen of photosynthetic origin in the oligotrophic oceans. Overall, the microbial gene capacity determines their adaptive ability to environmental variations in the ocean. A comprehensive microbial gene set from various ocean environments would facilitate to fully understand the connection between microbes and environmental variables.

The picocyanobacterium *Prochlorococcus* was the most abundant photosynthetic phytoplankton in the oligotrophic oceans, and contributes to the stability, resilience and function of marine ecosystem with high species diversity and a wide range of environmental adaptability (Biller et al., 2015; Farrant et al., 2016; Kent et al., 2016). In this study, the major *Prochlorococcus* clades in the Indian Ocean belonged to HLII ecotypes, which is in accordance with the sample origins from surface water with high light intensity. A previous study also showed that most of the Indian Ocean *Prochlorococcus* sequences were distantly related to the HLII *Prochlorococcus marinus* spp. such as MIT9215 and MIT9301, which were also detected in our samples (Díez et al., 2016). Furthermore, HLII bins also dominate the *Prochlorococcus* communities of the surface waters of the Red Sea based on analysis of the *rpoC1* sequences (Shibl et al., 2014). But the HLI, HLII, and HLVI clades of *Prochlorococcus* dominate in the surface water of the North Pacific Ocean by clustering the ITS sequences (Larkin et al., 2016). Phylogenetic analysis of 504 core genes across global ocean samples indicate that the clades HLIII and HLIV significantly dominate in the Equatorial Pacific Ocean, and the clad HLI is abundant in the California Current and South Atlantic Ocean (Kent et al., 2016). Therefore, the ecotype structure of *Prochlorococcus* differs among the oceanic regions. In this study, we constructed a phylogenetic tree based on 40 SCGs. This alternative method has been widely used in the clade classification of bacterial genomes (Hug et al., 2016; Mende et al., 2017). The phylogenetic clades showed the same accuracy as the ITS sequence (Biller et al., 2014) when we examined them using 25 fully sequenced reference genomes with known ecotypes. We also examined the accuracy of COG0172 in distinguishing species difference among 25 known ecotype genomes before exploring the global distribution of *Prochlorococcus*. It was demonstrated to be sufficient to discern the phylogenetic relation among *Prochlorococcus* genomes. The *Prochlorococcus* sequences from the global ocean could be divided into two clades (Moore et al., 1998; Rocap et al., 2002). Our study indicated that the HL clade occupied most of the sequences and showed less divergence, but the LL clades showed more branches with various phylogenetic distance. Similar observations were found in Yan’s study (Yan et al., 2018). At this point, most samples are sourced mainly from surface water and less from the deep ocean. The highly different phylogenetic distance of *Prochlorococcus* sequences from the deep ocean may reflect more diversity of *Prochlorococcus* genomes. These results illustrated that there was a considerable diversity of *Prochlorococcus* in the global ocean, but only a small portion can be cultured in the laboratory and a lot of unknown strains requires further excavation.

The RDA analysis showed that the *Prochlorococcus* abundance was positively associated with temperature and pH. Previous studies have shown that the HLI and HLII clades of *Prochlorococcus* present different optimal temperatures, indicating that temperature influences the distribution of *Prochlorococcus* (Zinser et al., 2007; Flombaum et al., 2013). The effect of pH on *Prochlorococcus* growth has been less studied, but it directly impacts the availability of bicarbonate for photosynthetic reaction, which may affect carbon fixation and cell growth. The abundance of *Prochlorococcus* was also significantly yet negatively correlated with nitrite concentration, suggesting the potential role of nitrite in *Prochlorococcus* growth.

The microbial functional genes involved in nitrogen metabolism indicated that bacteria in the oligotrophic ocean could assimilate N through different routes and they preferred urea, ammonia and nitrile, which need low energy to produce ammonia. The bacterial functional gene *nifH*, which is responsible for nitrogen fixation was absent in the surface water of the Indian Ocean and a similar phenomenon was also observed in the west Pacific Ocean (Li Y.-Y. et al., 2018), indicating that bacteria in the Indian Ocean surface waters lacked the ability to fix nitrogen. Ammonia assimilation of *Prochlorococcus* was explored in this study. The *Prochlorococcus* population can assimilate urea, ammonia, nitrite, nitroalkane, cyanate, and nitrile as nitrogen sources for cell growth, indicating that *Prochlorococcus* had evolved diverse adaptive strategies to ambient N deficiency in the oligotrophic Indian Ocean. The abundances of key enzymes involved in DON assimilation were higher than those involved in DIN assimilation in the *Prochlorococcus* population, implying that *Prochlorococcus* might prefer the organic N source in the oligotrophic Indian Ocean. The transcripts of key enzymes involved in the utilization of cyanate, urea and ammonia are also detected in the *Prochlorococcus* genomes under N stress condition (García-Fernández et al., 2004; Kamennaya and Post, 2011). To date, no *Prochlorococcus* isolate is able to utilize molecular N due to the lack of *nifH* genes, which concurs with our findings. The ability of nitrate assimilation is found only in a small portion of the *Prochlorococcus* genomes, such as SB and MIT0604, which is consistent with the findings of a recent study (Berube et al., 2019). They can grow on nitrate as the sole nitrogen source (Martiny et al., 2009; Berube et al., 2015). In our study, the gene encoding *NarB* catalyzing nitrate to nitrite was not detected in the *Prochlorococcus* population although it was found in 14 of the identified 54 *Prochlorococcus* genomes, indicating the limitation in assembling full sequences from metagenome data and the importance of isolation and identification of single bacterial strain from the natural environment, which would help us to unravel more novel and divergent metabolic pathways. Our study elaborated nitrogen assimilation pathways in the *Prochlorococcus* population and *Prochlorococcus* genomes in the Indian Ocean, and identified the differences in utilization of inorganic and organic nitrogen sources among different strains. This finding enables the potential to discover more unknown and uncultured strains from oceans.

It has been known for decades that most of the free-living bacteria can synthesize 20 kinds of amino acids, but some bacteria still have gaps in their biosynthesis pathways of amino acids (Price et al., 2018). Our study showed that *Prochlorococcus* could not make all amino acids by themselves based on their genetic content and might need to uptake some of them from the ambient environment instead. The gaps in *de novo* synthesis pathways of six different amino acids implied a special model for amino acid utilization in the *Prochlorococcus* population. Moreover, the distribution pattern of key enzymes involved in N and amino acid metabolism pathways in the metagenome of the *Prochlorococcus* population was validated by the *Prochlorococcus* genomes identified, demonstrating the reliability of our results. In general, our findings illustrated the potential capacity of *Prochlorococcus* to assimilate nitrogen in the Indian Ocean, which implied the adaptation of *Prochlorococcus* to ambient N deficiency in the oligotrophic ocean.

## Conclusion

The oligotrophic Indian Ocean is an understudied realm of the world’s oceans. This study comprehensively analyzed the microbial diversity and metabolic potential for N acquisition in the surface waters of the oligotrophic Indian Ocean using a metagenomic approach. Proteobacteria and Cyanobacteria dominated the microbial community but the functional composition of microbes exhibited a high level of gene diversity and functional redundancy across the Indian Ocean from the east to the west. Environmental factors such as temperature, phosphate, silicate, and pH played important roles in regulating microbial distribution in the Indian Ocean. Ammonium was an important nitrogen source for microbial community, while bacterial functional gene *nifH* was absent in the surface water of the Indian Ocean, indicating weak nitrogen fixation in the Indian Ocean and other potential ammonium origins, which needs further study. The predominant cyanobacterial taxa *Prochlorococcus* presented high diversity but a simple ecotype. Moreover, the *Prochlorococcus* evolved diverse adaptive strategies to ambient N deficiency in the oligotrophic ocean. Interestingly, the gaps for specific amino acid biosynthesis pathways existed in *Prochlorococcus*, demonstrating that there could be some alternative ways to acquire some essential amino, and the potential roles of *Prochlorococcus* in the biogeochemical cycle of amino acids. Overall, this study facilitated our understanding of microbes in the oligotrophic Indian Ocean, and serves as an important resource for gene capacity of microbes in future studies.

## Data Availability Statement

The 17 fastq files of SE reads were deposited in the CNSA (https://db.cngb.org/cnsa/) of CNGBdb with accession code CNP0000411. The 3 fastq files of PE reads were submitted to the NCBI SRA database with accession number PRJNA450884 (https://www.ncbi.nlm.nih.gov/bioproject/PRJNA450884).

## Author Contributions

D-ZW, Y-YW, TJ, and XL established the concept of the study. YBG, Z-XX, and L-FK collected and processed the samples. Y-YW, S-LL, G-LL, and S-HG performed the bioinformatics analyses. Y-YW, S-LL, and P-FZ performed the data analysis. Y-YW wrote the draft. D-ZW, HL, LG, M-LS, S-LL, S-KS, and G-YF revised and edited the manuscript. All the authors have discussed the results, read and approved the contents of the manuscript.

## Conflict of Interest

Y-YW, S-LL, G-LL, TJ, HL, G-YF, S-SL, S-KS, S-HG, P-FZ, and XL was employed by the institute of BGI-Shenzhen. The remaining authors declare that the research was conducted in the absence of any commercial or financial relationships that could be construed as a potential conflict of interest.

## Footnotes

1 http://www.comra.org/

## Supplementary Material

Supplementary Figure 1 The workflow of experiment and data analysis. A total of 17 samples were collected in the Indian Ocean and metagenome sequencing was performed on BGISEQ and HiSeq platforms, separately.

Supplementary Figure 2 Assessment of the efficiency of gene identification between SE reads mapping and PE reads mapping from the same three samples. Green and orange represents the novel genes in OM-RGC mapped by SE and PE reads, respectively. Yellow represents the genes shared by both.

Supplementary Figure 3 Microbial taxonomic composition and predominant bacterial phylum in the Indian Ocean surface water. **(A)** Taxonomic composition. **(B)** The predominant bacterial phylum. Others represented bacterial abundance lower than 0.1%.

Supplementary Figure 4 The heat map of physicochemical properties from oceanic samples.

Supplementary Figure 5 The distribution pattern of the detected *Prochlorococcus* genomes among sampling stations of the Indian Ocean.
