# Supplementary figures and images for: Metagenomic Analysis Reveals Microbial Community Structure and Metabolic Potential for Nitrogen Acquisition in the Oligotrophic Surface Water of the Indian Ocean
Source: Front Microbiol. 2021 Feb 18;12:518865. doi: 10.3389/fmicb.2021.518865 (PMC7935530; doi:10.3389/fmicb.2021.518865)

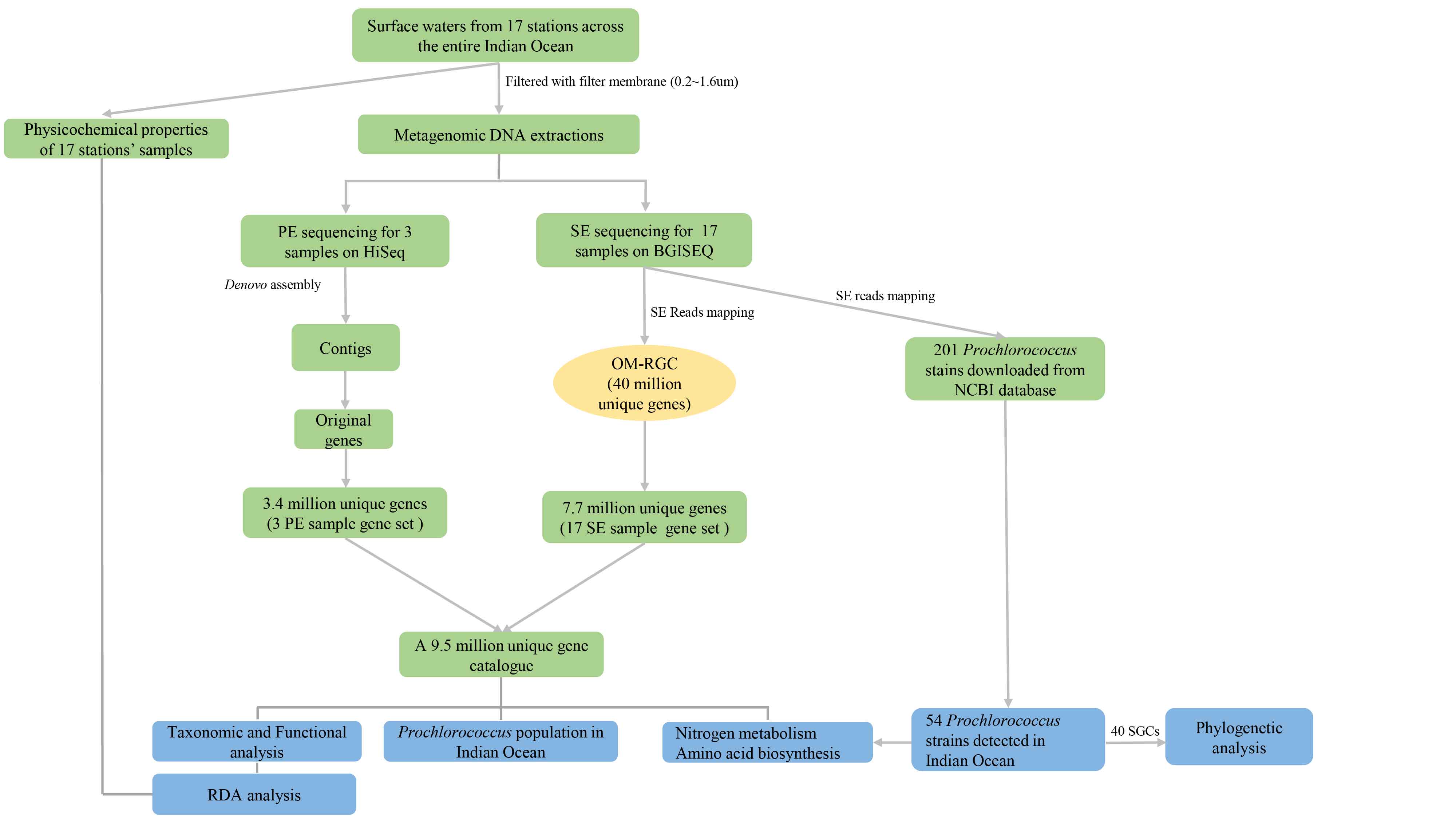

Supplement: Supplementary Figure 1 — The workflow of experiment and data analysis. A total of 17 samples were collected in the Indian Ocean and metagenome sequencing was performed on BGISEQ and HiSeq platforms, separately. [file Image_1.JPEG]

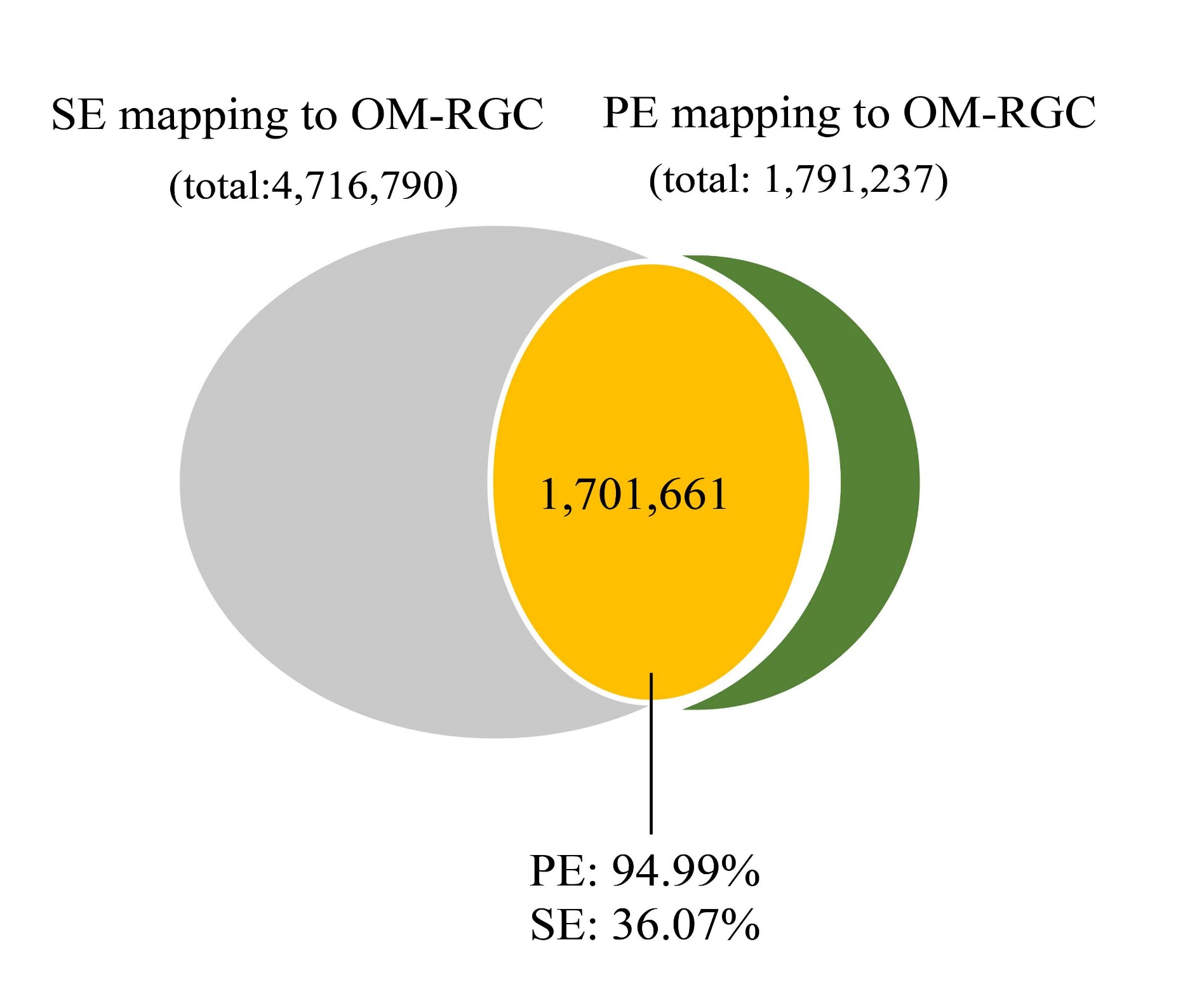

Supplement: Supplementary Figure 2 — Assessment of the efficiency of gene identification between SE reads mapping and PE reads mapping from the same three samples. Green and orange represents the novel genes in OM-RGC mapped by SE and PE reads, respectively. Yellow represents the genes shared by both. [file Image_2.JPEG]

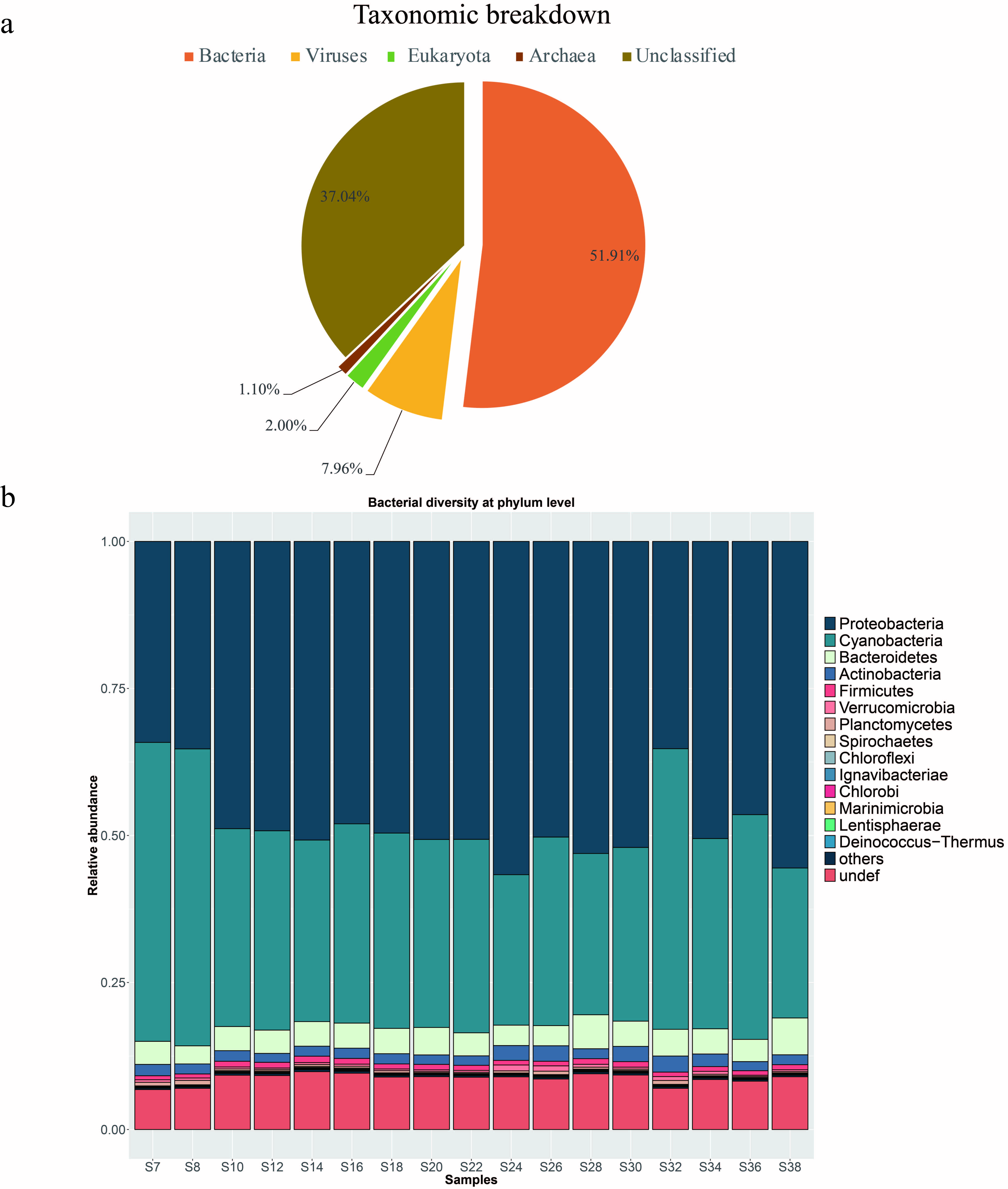

Supplement: Supplementary Figure 3 — Microbial taxonomic composition and predominant bacterial phylum in the Indian Ocean surface water. (A) Taxonomic composition. (B) The predominant bacterial phylum. Others represented bacterial abundance lower than 0.1%. [file Image_3.JPEG]

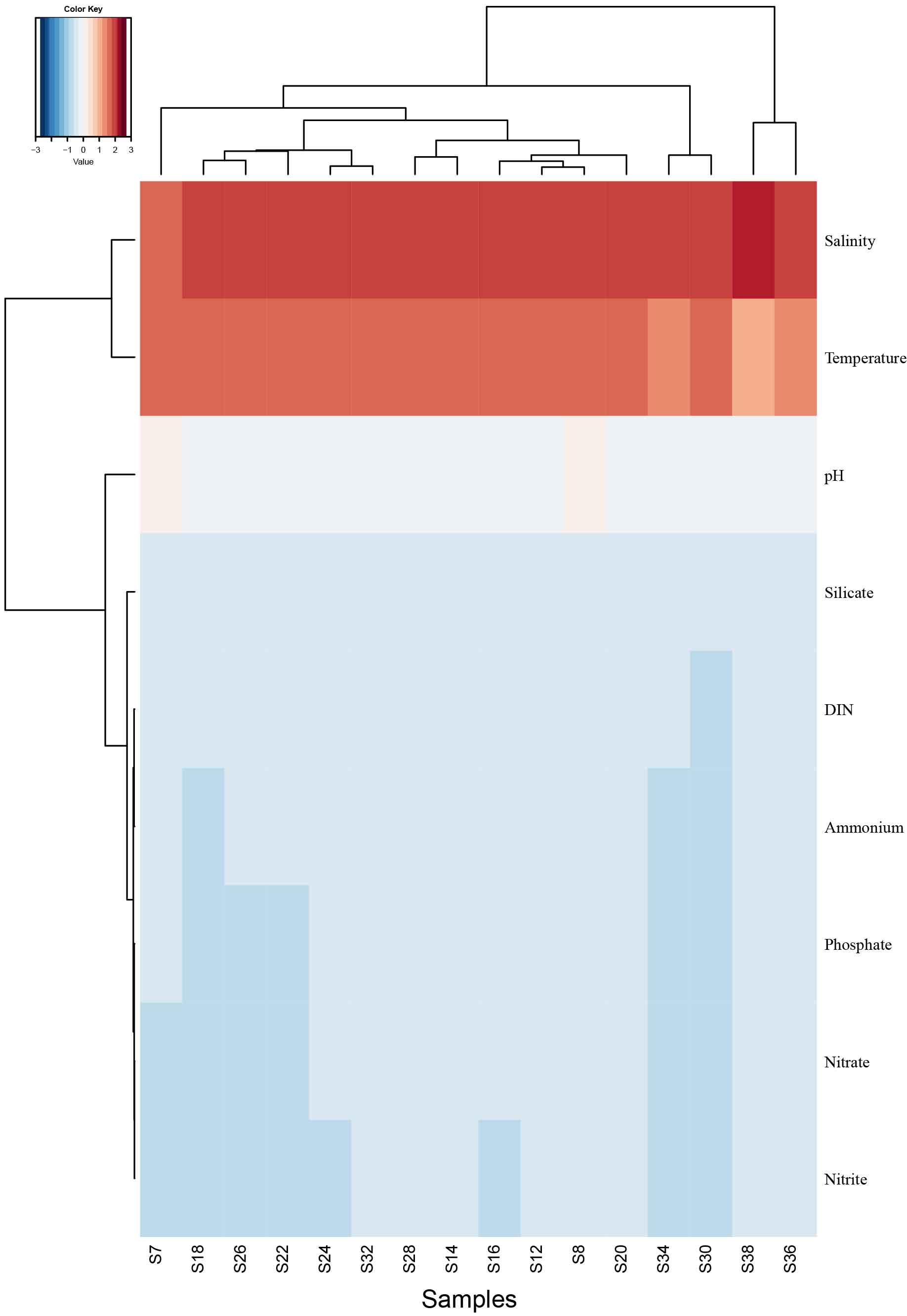

Supplement: Supplementary Figure 4 — The heat map of physicochemical properties from oceanic samples. [file Image_4.JPEG]

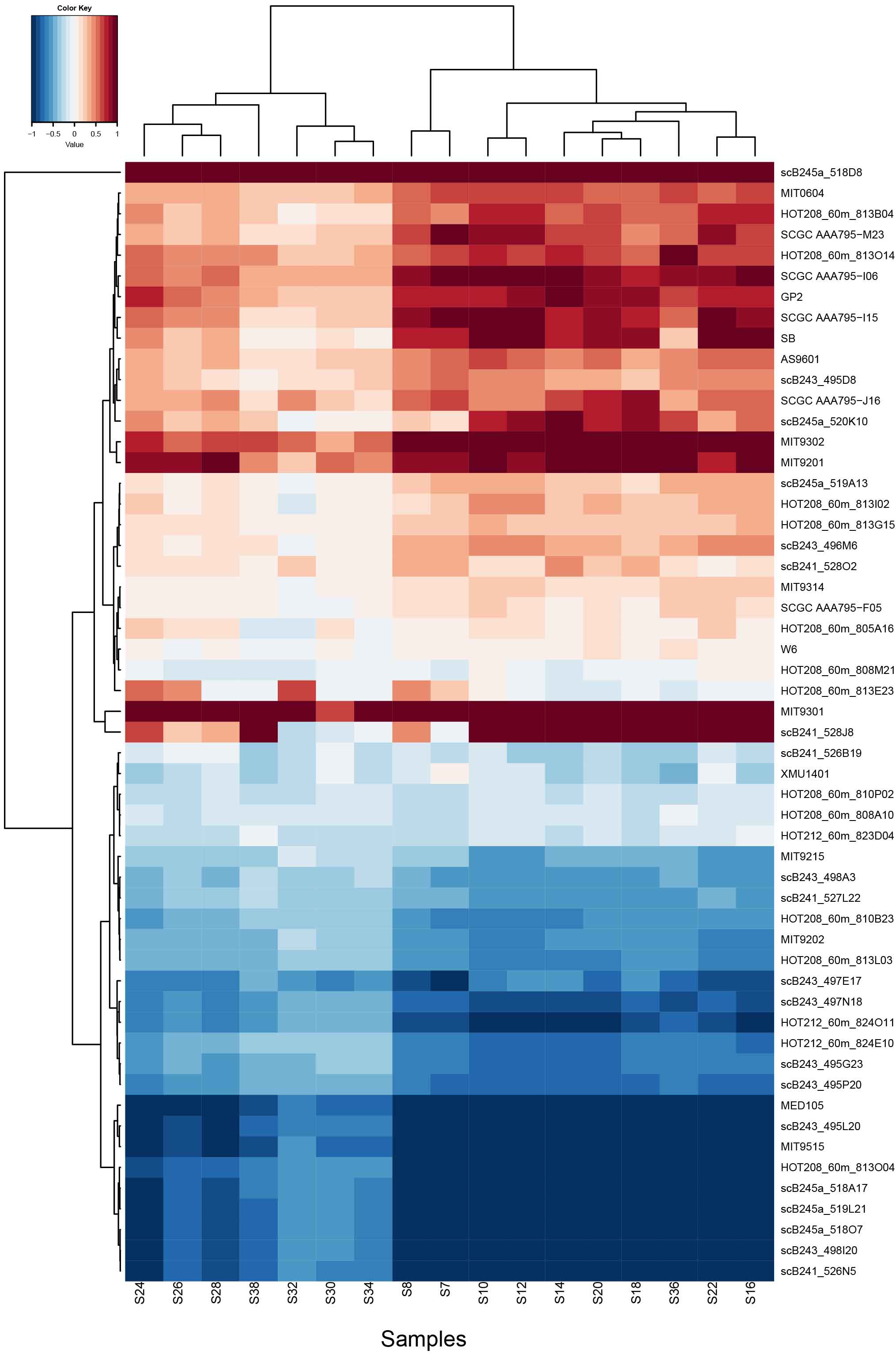

Supplement: Supplementary Figure 5 — The distribution pattern of the detected Prochlorococcus genomes among sampling stations of the Indian Ocean. [file Image_5.JPEG]
